# Interannual Differences for Sea Turtles Bycatch in Spanish Longliners from Western Mediterranean Sea

**DOI:** 10.1155/2014/861396

**Published:** 2014-02-10

**Authors:** José C. Báez, David Macías, Salvador García-Barcelona, Raimundo Real

**Affiliations:** ^1^Instituto Español de Oceanografía, Centro Oceanográfico de Málaga, Puerto Pesquero s/n, Fuengirola, 29640 Málaga, Spain; ^2^Biogeography, Diversity, and Conservation Research Team, Department of Animal Biology, University of Málaga, 29071 Málaga, Spain

## Abstract

Recent studies showed that regional abundance of loggerhead and leatherback turtles could oscillate interannually according to oceanographic and climatic conditions. The Western Mediterranean is an important fishing area for the Spanish drifting longline fleet, which mainly targets swordfish, bluefin tuna, and albacore. Due to the spatial overlapping in fishing activity and turtle distribution, there is an increasing sea turtle conservation concern. The main goal of this study is to analyse the interannual bycatch of loggerhead and leatherback turtles by the Spanish Mediterranean longline fishery and to test the relationship between the total turtle by-catch of this fishery and the North Atlantic Oscillation (NAO). During the 14 years covered in this study, the number of sea turtle bycatches was 3,940 loggerhead turtles and 8 leatherback turtles, 0.499 loggerhead turtles/1000 hooks and 0.001014 leatherback turtles/1000 hooks. In the case of the loggerhead turtle the positive phase of the NAO favours an increase of loggerhead turtles in the Western Mediterranean Sea. However, in the case of leatherback turtle the negative phase of the NAO favours the presence of leatherback turtle. This contraposition could be related to the different ecophysiological response of both species during their migration cycle.

## 1. Introduction

During the last two decades, our understanding of sea turtle conservation biology has increased significantly. It is now widely believed that the major threat for the sea turtles is caused by diverse anthropogenic activities [1, 2]. Early evidence suggests that longline fishing is a major source of mortality in the Mediterranean Sea [3].

The endangered loggerhead turtle (*Caretta caretta*) is the most common sea turtle species in the Mediterranean Sea, where it utilises nesting beaches which are mainly located in the eastern basin. Annually, hundreds of juvenile loggerhead turtles, born on the beaches of both the North Atlantic [4, 5] and Mediterranean, are concentrated around the feeding grounds in the Western Mediterranean, mainly in waters around the Balearic Islands [6–8]. Revelles et al. [9] suggest the existence of a permeable barrier north of the Balearic Islands that divides the Northwestern Mediterranean and the Southern Mediterranean basin which affects the distribution of loggerhead turtles. This barrier could have an impact on the distribution of both migrant turtles from the Atlantic and the Eastern Mediterranean. In any case, many juveniles born in the Atlantic remain in the Mediterranean until they reach the minimum size to overcome the flow of Atlantic waters entering in the Mediterranean through the Strait of Gibraltar [10, 11].

The Western Mediterranean is an important fishing area for the Spanish drifting longline fleet which mainly targets swordfish (*Xiphias gladius*), bluefin tuna (*Thunnus thynnus*) and albacore (*Thunnus alalunga*) [12, 13]. Due to the spatial overlap in fishing activity and loggerhead turtle distribution, each year tens of thousands of loggerhead turtle bycatches occur [3, 14]. There are several studies about the loggerhead turtle bycatch by the Spanish longline fishery in the Mediterranean [15–23]. The main results of these early studies are that gear configurations (e.g., hook and bait type) by Spanish Mediterranean longliners can determine both- bycatch frequency [15] and catch selectivity of sizeclasses [19, 23]. In addition, Báez et al. [18, 22] concluded that the loggerhead turtles bycatches were spatially structured only according to mean distance to the coast. Moreover, the bycatches of loggerhead turtles increased significantly within those longline sets hauling during daylight [17].

However, there are no studies of the interannual loggerhead bycatch by Spanish fisheries. In this context, Tomás et al. [24], using indirect data from strandings, concluded that loggerhead turtle bycatch may have decreased in the last years. However, there are few studies about interannual variability of bycatch of loggerhead by Spanish fisheries.

Leatherback sea turtle (*Dermochelys coriacea*) is listed as Vulnerable on the UICN red list [25] and is the second most common chelonian migratory species in the Mediterranean, where nesting beaches do not exist [26]. Leatherback turtles are also caught accidentally by Spanish longliners but show low levels of bycatch per unit of fishing effort [1, 27].

Sea turtles present a long-distance migration of juveniles resulting in broad-scale dispersion of these juvenile stages. In this context, some studies concluded that abundance of sea turtles (both loggerhead and leatherback turtles) could oscillate interannually according to oceanographic and climatic conditions [28, 29].

The main goal of this study is to analyse interannual variability in data of bycatch of loggerhead and leatherback turtles from Spanish Mediterranean longline fishery and subsequently test the possible relationship between the CPUE (catch per unit effort, measured in thousands of hooks) of these turtles and the atmospheric oscillations.

## 2. Material and Methods

### 2.1. Data Collection

**Figure 1:**
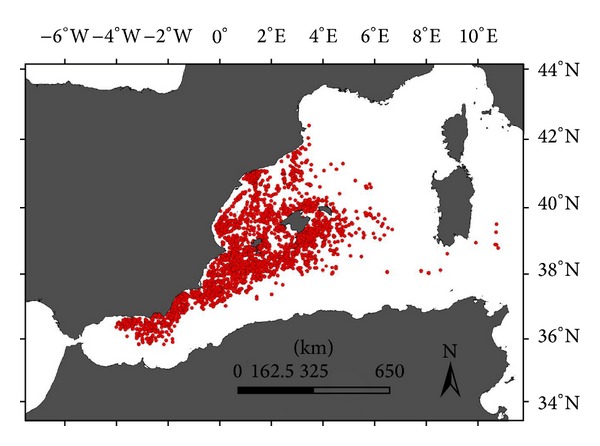
Fisheries effort distributions of Spanish surface longline fleet for entire study period.

Data were collected by IEO observers on board Spanish longliners between 1999 and 2012. The fishing ground was mainly limited to the Levantine-Balearic, Algerian, and Alborán Sea regions (Figure 1); the drifting surface longline gears reported within this area by the International Commission for the Conservation of Atlantic Tunas (ICCAT) include drifting surface longline targeting albacore (LLALB), surface longliners targeting bluefin tuna (LLJAP), and traditional drifting surface longliners targeting swordfish (LLHB). Briefly, the principal differences between the three different longline gears are as follows.

LLJAP: this is a monofilament longline used exclusively during the months of May, June, and the first half of July. The bait is almost always squid (*Illex* sp.) bigger than 500 g. LLJAP typically uses a C-shaped hook, and its number per set does not exceed 1200, and these are laid in a circle.

LLHB: this gear is used throughout the year. The number of hooks is between 1,500 and 4,000 and these are usually baited with mackerel (*Scomber* sp.) and squid (*Illex* sp.). The dimensions and forms of the hooks used are J-shaped Mustad-2 (approximately 7.5 × 2.5 cm).

LLALB: this is the shallowest longline gear. Both the size of the hook and the thickness and length of the fishing lines are lower than other longlines, usually J-shaped Mustad number 5 (approximately 5 × 2 cm). Between 2,000 and 7,000 hooks are set and the bait used is sardine (*Sardina pilchardus*). This gear is used mainly from July to October.

The Spanish longline fleet licensed for surface longline targeting highly migratory species, such as tuna and swordfish, consists of an average of 89 vessels per year for the studied period, with a vessel length ranging from 12 to 27 m. A total of 3,412 fishing operations were observed from January to December, during the years 1999 to 2012, which represent 7,889,711 observed hooks. Camiñas et al. [15], Báez et al. [18, 21, 22], and García-Barcelona et al. [13, 30] described the Spanish longline fishery in detail and reviewed the data collection performed by the IEO onboard observer program.

### 2.2. Climatic Index

The most important mechanism responsible for interannual climate variability in South-West Europe is the North Atlantic Oscillation (NAO) [31, 32]. The NAO reflects fluctuations in atmospheric pressure at sea level between the Icelandic Low and the Azores High. The NAO is associated with many meteorological variations in the North Atlantic region, affecting wind speed and direction and differences in temperature and rainfall. The North Atlantic Oscillation (NAO) usefully explains ecological fluctuations [33] affecting the North Atlantic migrating fauna [28]. We used teleconnection patterns in the atmospheric circulation for pressure anomalies based on the normalized pressure to 500 hPa for NAO and height anomalies at 1000 hPa for AO [34].

Monthly NAO index values were taken from the website of the National Oceanic and Atmospheric Administration (NOAA website: Available at http://www.cpc.noaa.gov/products/precip/CWlink/pna/nao_index.html. Accessed May 15, 2013).

The NAO presents strong interannual and intraannual variability [31, 32], with a strong NAO pattern in cold seasons, primarily from November to March. However, given that a previous paper [28] found a significant relationship between the abundance of sea turtles and the mean annual NAO of the previous year, we related relative abundance values of turtle bycatches with the mean annual NAO of the previous year (NAOpy).

### 2.3. Statistical Analysis

Given that the mean annual leatherback turtle bycatches varied by about 0.57 turtles during the study period, it offers little possibility for sound statistical analysis to look for patterns or trends. However, a probability analysis may be introduced by taking a year at random and calculating the probability of observing one leatherback turtle bycatch or not.

Binary logistic regression is widely used for establishing relationships between environmental independent variables and the probability of response of target variables (e.g., [35]). We used a binary logistic regression to estimate the probability to obtain in a particular year observed at least one leatherback turtle bycatches in function to average NAOpy.

As we commented above, Revelles et al. [9] suggest the existence of a permeable barrier north of the Balearic Islands that affects the distribution of loggerhead turtles. Moreover, the probability of capture of one loggerhead turtle in longline gear as a whole is susceptible to variations, regardless of the total fishing effort, depending on the configuration of the gear used. So the CPUEs for turtles should be compared independently for each gear (LLHB, LLALB and LLJAP) in order to estimate total turtle bycatch. At the same time LLHB presented a greater number of direct turtle bycatch. For these reason, we analysed the CPUE of loggerhead turtle (CPUEt) from south of 40°N for LLHB versus the NAOpy. In this case, we calculated the probability obtaining a CPUEt of a particular year greater than the average CPUEt for all the years with catches. Consequently, we assigned the value 1 when the CPUEt of a particular year was greater than the mean CPUEt for all the years with catches pooled together, while we assigned the value 0 when the CPUEt of a particular year was lower than the mean CPUEt.

To evaluate the models we assessed their goodness-of-fit and discrimination capacity. The model goodness-of-fit was assessed by means of the Hosmer and Lemeshow test. We evaluated the discrimination capacity of our model with the area under the receiving operating characteristic curve (AUC) [36, 37].

As logistic regression is sensitive to the 1/0 frequencies ratio [36], we used the favourability function (*F*) proposed by Real et al. [35] to adjust the model according to this ratio. Favourability was calculated from the probability obtained from logistic regression as follows:

(1) $$\begin{array}{c} F=\frac{\left[P/\left(1-P\right)\right]}{\left[\left(n_{1}/n_{0}\right)+\left(P/\left[1-P\right]\right)\right]}, \end{array}$$

where *P* is the probability from logistic regression, *n*_1_ is the number of years with positive bycatches event for loggerhead or leatherback turtle (previously defined), and *n*_0_ is the number of years with negative bycatches event for loggerhead or leatherback turtle (previously defined).

We used *F* to assess the atmospheric oscillation conditions that favoured the bycatches event for loggerhead or leatherback turtle. We then compared the correct classification rate of the models for favourable and clearly unfavourable years.

The main advantages of the favourability function are (according to Real et al. [35] and Acevedo and Real [38]).

1. The favourability function adjusts the model to inform about the degree to which the environmental conditions favour the event, regardless of the 1/0 frequencies ratio.
2. The threshold 0.5 from favourability model is easier to interpret, as it indicates neutral environmental conditions, that is, neither favourable nor unfavourable.
3. The favourability function yielded graphic models which were easier to interpret. Moreover, the favourability model allows performing a clarifying graphical comparison between two different states from one same qualitative variable.

## 3. Results

**Figure 2:**
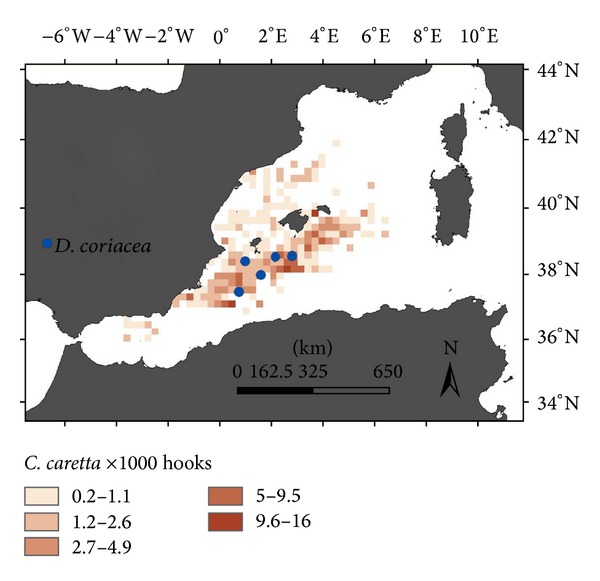
Distribution of bycatches of loggerhead (coloured scale) and leatherback turtle (blue circle).

During the 14 years covered in this study, the number of sea turtles caught as bycatch was 3,940 loggerhead turtles and 8 leatherback turtles, 0.499 loggerhead turtles/1000 hooks and 0.001014 leatherback turtles/1000 hooks (Figure 2). In Table 1 we show the frequency of turtle bycatches observed per year.

Statistical analysis of the data indicates that CPUEt presents significant interannual differences (Table 1) (*χ* ^2^ = 2136.85; df = 13; *P* < 0.0001). Similarly, we observed a lack of leatherback turtle bycatches in ten years, with only four years with leatherback turtle bycatches.

When we analysed the probability of obtaining at least one leatherback turtle bycatch in a particular year in function of the average NAOpy, we obtained a significant logistic regression model (*χ* ^2^ = 4.512, df = 1, *P* = 0.034) with no significant difference (Hosmer and Lemeshow test, *χ* ^2^ = 7.954, df = 8, *P* = 0.438) between predicted and observed values, with a good discrimination capacity (AUC = 0.833). The parameter for NAOpy was negative, so the higher the annual NAOpy index for a particular year, the lower the probability of getting an annual bycatch of at least one leatherback turtle. The logit function (*y*) derived from logistic regression presents the form:

(2) $$\begin{array}{c} y_{\text{leatherback}}=-1.376-5.783*\text{NAOpy}. \end{array}$$

For the loggerhead turtle, when we analysed the effect of NAOpy on the probability of catch of a greater number of turtles (with the LLHB gear at south of 40°N) we obtained a significant logistic regression model (*χ* ^2^ = 6.422, df = 1, *P* = 0.011) with no significant difference (Hosmer and Lemeshow *P* = 0.786) between predicted and observed values, with acceptable discrimination capacity (AUC = 0.85). The parameter for NAOpy was positive, so the higher the annual NAOpy index for a particular year, the higher the probability of obtaining a greater number of loggerhead turtles than the average of all years pooled together. The logit function (*y*) derived from logistic regression presents the form:

(3) $$\begin{array}{c} y_{\text{loggerhead}}=-0.742+7.273*\text{NAOpy}. \end{array}$$

**Figure 3:**
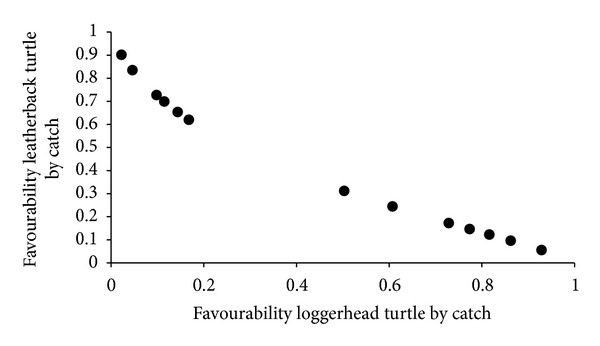
Favourability of obtaining at least one leatherback turtle bycatch in a particular year in function of the average NAO in previous year versus the favourability to obtain a major number of loggerhead turtles bycatches (in the LLHB gear fisheries south of 40°N) in function of the average NAO in the previous year.

In Figure 3, we show the favourability of bycatch of at least one leatherback turtle versus the favourability of bycatch of a greater number of loggerhead turtles according to NAOpy conditions.

## 4. Discussion

In both turtle species studied, the NAOpy in the previous year plays an important role in their migration. However, while in the case of the loggerhead turtle the positive phase of the NAOpy favours its abundance increment in the Western Mediterranean, in the case of leatherback turtle the negative phase of the NAOpy favours its presence. This contraposition could be related to the different ecophysiological response of both species during their migration cycle. Recent studies suggest an important role of currents and storms in dispersal of loggerhead turtles [39]. Thus, the arrival of juvenile loggerhead turtles into the Mediterranean through the Strait of Gibraltar seems to be motivated by a combination of favourable currents and storms, rather than preestablished migration routes. This could explain the interannual oscillation in the abundance of loggerhead turtles in the Mediterranean, which in turn could lead to an increased number of strandings and catches, according to the present results and previous studies [28].

On the other hand, leatherback turtle is a predator of mesoplankton (i.e., jellyfish). For this reason many authors have noted that the migration of this species could be fitted to the intensive search for food [40, 41]. Thus, the leatherback turtle is more efficient in finding productive areas than the loggerhead [40, 41]. In this context, negative NAO phase has been implicated as drivers for induced blooms along the coast of the Iberian Peninsula [42–44]. On the Galician coast (Northwest Spain), this effect should lead to increased abundance of phytoplankton (e.g., *Gymnodinium catenatum*), strong vertical migratory species capable of utilising deeper remineralised nutrients from the decomposition of postbloom sedimented materials [42–44]. Therefore, a negative phase of the NAOpy could imply increased runoff and increased contribution of land-based materials to the sea; this increased nutrient could increase plankton productivity, which in turn attracts jellyfishes, and they in turn attract the leatherback turtles.

### 4.1. Implications for Surface Longline Fishery Management

Previous studies showed differential bycatch frequency and size differentiation in loggerhead turtles as a function of surface longline gear type in the Western Mediterranean Sea [23]. Thus, surface longline targeting albacore (LLALB) using smaller hooks tends to capture smaller loggerheads and has the highest CPUEt, whereas other longlines, such as surface longline targeting bluefin tuna (LLJAP) and traditional surface longline targeting swordfish (LLHB), using larger hooks tend to select the larger animals; moreover, LLHB had the lowest CPUEt.

Currently, the use of a particular gear is determined by the fishery regulations, market dynamics, and fish prices. According to recommendations from* The International Commission for the Conservation of Atlantic Tunas* (ICCAT), the current legislation on longline contemplates a fishing moratorium during October (ORDEN/APA/254/2008, BOE n. 33 2008; ORDEN/ARM/817/2009, BOE n. 79 2009) and other closure of fishing activity during February and March (*Resolución de 19 de febrero de 2013*, BOE n. 46 2013), which could be beneficial for the swordfish stock. In this context, because we know the NAO in advance, we suggest alternating the gear type each year and removing temporary closures in function of previous climatic conditions and previsions. During the years subsequent to a negative NAO phase, a preventive cessation of activity for the LLALB could avoid an important bycatch of loggerhead juveniles, but the use of the LLHB and LLJAP could not affect significantly the bycatch of the largest loggerhead turtles. On the contrary, for the years subsequent to each positive NAO phase a preventive closing of activity for the LLHB could avoid high CPUEt of mature turtles. Thus, we could reduce the total juvenile of loggerhead turtle bycatch during the preventive closing of activity for the LLALB and in other years avoid the mature bycatch of loggerhead turtles during the preventive closure of activity for the LLHB and LLJAP.

Nevertheless, the adoption of these recommendations implies a greater control of the fishery and a changing of regulations in a short time (year to year) really difficult to implement for both fishermen and administration. However, we believe that a global fisheries management scheme which implicates both ecological and temporal perspectives is necessary.

## Figures and Tables

**Table 1:** Observed hooks and turtle bycatch per year during the study period. Expected loggerhead values were calculated according to the number of hooks, and thus the expected loggerhead is (total loggerhead turtles observed in all the study period ∗ the total hooks observed in the year)/total hooks observed in all the study period.

Year	Hooks ∗ 1000	Loggerhead	Leatherback	Expected loggerhead
1999	830.87	466	1	414.92
2000	1196.996	1098	0	597.76
2001	705.766	347	0	352.45
2002	486.707	114	3	243.054
2003	345.209	370	1	172.39
2004	336.765	467	0	168.18
2005	112.71	45	0	56.29
2006	484.933	322	0	242.16
2007	307.654	93	0	153.64
2008	323.872	32	0	161.74
2009	394.28	71	0	196.9
2010	513.453	338	3	256.41
2011	854.183	16	0	426.57
2012	996.313	161	0	497.54

Total	7889.711	3940	8	—
